# Macroscopic model and statistical model to characterize electromagnetic information of a digital coding metasurface

**DOI:** 10.1093/nsr/nwad299

**Published:** 2023-11-29

**Authors:** Rui Wen Shao, Jun Wei Wu, Zheng Xing Wang, Hui Xu, Han Qing Yang, Qiang Cheng, Tie Jun Cui

**Affiliations:** Institute of Electromagnetic Space, Southeast University, Nanjing 210096, China; State Key Laboratory of Millimeter Waves, Southeast University, Nanjing 210096, China; Institute of Electromagnetic Space, Southeast University, Nanjing 210096, China; State Key Laboratory of Millimeter Waves, Southeast University, Nanjing 210096, China; Peng Cheng Laboratory, Shenzhen 518055, China; Pazhou Laboratory (Huangpu), Guangzhou 510555, China; Institute of Electromagnetic Space, Southeast University, Nanjing 210096, China; State Key Laboratory of Millimeter Waves, Southeast University, Nanjing 210096, China; Institute of Electromagnetic Space, Southeast University, Nanjing 210096, China; State Key Laboratory of Millimeter Waves, Southeast University, Nanjing 210096, China; Institute of Electromagnetic Space, Southeast University, Nanjing 210096, China; State Key Laboratory of Millimeter Waves, Southeast University, Nanjing 210096, China; Institute of Electromagnetic Space, Southeast University, Nanjing 210096, China; State Key Laboratory of Millimeter Waves, Southeast University, Nanjing 210096, China; Institute of Electromagnetic Space, Southeast University, Nanjing 210096, China; State Key Laboratory of Millimeter Waves, Southeast University, Nanjing 210096, China; Peng Cheng Laboratory, Shenzhen 518055, China; Pazhou Laboratory (Huangpu), Guangzhou 510555, China

**Keywords:** electromagnetic information theory, current entropy, current space, digital coding metasurface, macroscopic model, statistical model

## Abstract

A digital coding metasurface is a platform connecting the digital space and electromagnetic wave space, and has therefore gained much attention due to its intriguing value in reshaping wireless channels and realizing new communication architectures. Correspondingly, there is an urgent need for electromagnetic information theory that reveals the upper limit of communication capacity and supports the accurate design of metasurface-based communication systems. To this end, we propose a macroscopic model and a statistical model of the digital coding metasurface. The macroscopic model uniformly accommodates both digital and electromagnetic aspects of the meta-atoms and predicts all possible scattered fields of the digital coding metasurface based on a small number of simulations or measurements. Full-wave simulations and experimental results show that the macroscopic model is feasible and accurate. A statistical model is further proposed to correlate the mutual coupling between meta-atoms with covariance and to calculate the entropy of the equivalent currents of digital coding metasurface. These two models can help reconfigurable intelligent surfaces achieve more accurate beamforming and channel estimation, and thus improve signal power and coverage. Moreover, the models will encourage the creation of a precoding codebook in metasurface-based direct digital modulation systems, with the aim of approaching the upper limit of channel capacity. With these two models, the concepts of current space and current entropy, as well as the analysis of information loss from the coding space to wave space, is established for the first time, helping to bridge the gap between the digital world and the physical world, and advancing developments of electromagnetic information theory and new-architecture wireless systems.

## Teaser

The author proposes a novel and accurate macroscopic model bridging the codes and scattered waves of the metasurface, and further develops a statistical model to characterize the information capacity of the metasurface.

## INTRODUCTION

Metamaterials are artificial structures exhibiting properties and functions not found in nature, such as negative refraction [1], perfect imaging [2,3] and invisibility cloaking [4]. As the two-dimensional (2D) form of metamaterials, metasurfaces have presented intriguing capabilities in tailoring electromagnetic (EM) waves in microwave and millimeter waves at low costs [5–7]. A metasurface in the optical band also shows many potential applications, including optical-inspired broadband absorbers [8], breaking the fundamental limit of traditional interference lithography [9] and merging the incompatible diffuse reflection and undistorted transmission [10]. The subwavelength meta-atoms can modulate the amplitudes and phases of incident EM waves, thus manipulating the scattered wavefronts with great flexibility. Recently, digital coding and information metasurfaces have attracted a lot of attention in both EM and wireless communication communities [11–27]. The elements of digital and information metasurfaces work in a discrete manner and exhibit a finite number of states [11–13]. For example, a 1-bit metasurface element gives rise to EM fields with opposite phases, which are denoted by digital states ‘0’ and ‘1’, respectively. The application of digital and information metasurfaces can be roughly divided into two types. The first type are known as reconfigurable intelligent surfaces (RISs) [14,15] or intelligent reflecting surfaces (IRSs) [16,17], wherein the metasurfaces act as anomalous reflectors and boost signal levels in the direction of interest. Several prototypes of RIS-assisted communication systems have been developed to demonstrate their ability to control wireless channels and enhance signal powers [18,19]. The utilization of RISs will help reduce the construction cost of future wireless communication systems and networks [14].

The second type is metasurface-based direct digital modulation (DDM) [20], in which the states of meta-atoms are rapidly changed according to the time-domain signals, and the scattered waves are a mixture of EM waves and baseband information [21]. Such a technology does not rely on traditional digital–analog converters and mixers, thus helping reduce complexity and increase flexibility. A variety of DDM systems have been reported [21–23], such as space-frequency-division multiplexing, multiple-input multiple-output (MIMO) and multi-stream signals separation wireless communications based on space-time-coding metasurfaces [24–26]. Information entropy and theory of digital metasurfaces have also been investigated to quantitatively evaluate their modulation and transmission capabilities [27,28].

In practical applications of RIS and DDM systems, an exact and uniform model is needed to determine their array sizes, geometry configurations, coding patterns and other key parameters. Various models have been proposed [29–37], but they have not taken the physical characteristics of metasurfaces fully into account. The existing modeling methods can be broadly classified into three types.

The first type is the full-wave method, wherein the metasurface is modeled accurately and Maxwell's equation is solved strictly via numerical methods [38–47]. However, simulating large-scale digital metasurfaces and RISs is computationally prohibitive due to the huge number of meta-atoms and coding states. In view of this, the full-wave method is reliable but not friendly to engineering applications. The second type of method treats the metasurface as an aperture composed of discrete currents [45–47]. The meta-atoms are assumed to exhibit the same current pattern but different amplitudes and phases depending on the coding states. The simplest current pattern is the point source located in the center of the meta-atom, whose far-field is believed to be omnidirectional or cosine [48–53]. Nevertheless, this model neglects the effects of incidence angle and element size on the radiation pattern. In fact, the meta-atoms respond sensitively to different incidence angles, and their gains vary with the electric sizes. In [54], the meta-atoms are approximated as rectangular metal sheets of the same size, whose responses under different incidence angles have an analytical expression. However, the digital meta-atoms are usually composed of both dielectrics and metals, whose equivalent currents are significantly different from those of pure metal sheets. In addition, none of these methods takes the mutual coupling between adjacent meta-atoms into consideration, giving rise to errors in the results. Therefore, this type of method features high efficiency but low accuracy. In the third type of method, the metasurface is regarded as a multi-port device, and the microwave network theory is used to analyze the scattered fields [55,56]. This type of method can effectively characterize the mutual coupling of meta-atoms, but the acquisition of network parameters still relies on the above-mentioned two methods. Thus, this method is still caught in a trade-off between accuracy and efficiency.

In fact, an accurate macroscopic model of the digital metasurface is the key to ensuring the validity of channel modeling and further applications, such as channel estimations and beamforming. Hence, it is not only a starting point for element design but also a basis for the overall plan of metasurface-based communication systems. More importantly, the current research and application of digital metasurfaces urgently require the development of EM information theory to sew up the gap between digital information and EM physics, and the macroscopic model is a key component of EM information theory. Meanwhile, none of the existing models has provided uniform and accurate descriptions of digital and EM information. The over-idealized modeling method cannot consider mutual coupling and other factors when analyzing the information capacity of the digital metasurface. The resonance modes of the metasurface element in two coding states are different, therefore they cannot be considered as identical patterns with 180° phase difference. In addition, the scattering of meta-atoms will be affected by the coding states of adjacent elements. All these factors mean that metasurfaces in practical applications are far from the ideal model, which eventually leads to inaccurate results in information capacity analysis.

Here, we propose a macroscopic model of the digital metasurfaces, which is uniform, efficient and accurate. The model is based on rigorous mathematical and physical analysis, and innovatively introduces the concept of current space. By including high-order current patterns in equivalent networks and taking the mutual coupling of meta-atoms into consideration, a mapping between the digital codes and EM fields is established. Based on the model, we achieve a balance between efficiency and accuracy in characterizing the meta-atoms and can predict the scattered fields of the digital metasurfaces working in any coding state. Furthermore, we propose a novel statistical model of the current and link the mutual coupling in EM information theory with the covariance in statistics. Based on this model, we calculate the joint probability density function of the equivalent currents on the metasurface aperture, and further quantify the information capacity by introducing the concept of current entropy. Current entropy represents the information of the currents on the metasurface. The input of the metasurface is digital code and its output is current, when it works as a transmitter. Therefore, contrary to the coding entropy in [27,28], the current entropy can characterize the transmitted information of a metasurface more accurately.

In this paper, we propose a macroscopic model and a statistical model of the digital coding metasurface. The macroscopic model can accurately calculate the scattered fields of RISs. This model will ensure the accuracy of channel estimation and the performance of beamforming, thus improving the power and coverage of transmitted signals. The statistical model provides an effective method of calculating the covariance matrix between the elements of the DDM transmitter, which lays the foundation for designing a precoding codebook and helps approximate the upper limit of channel capacity. The proposed two models fulfill the demands of the practical engineering designs of the digital metasurface, and contribute to the rapid development of EM information theory and related wireless systems.

## MACROSCOPIC MODEL BRIDGING DIGITAL CODES AND ELECTROMAGNETIC FIELDS

### Introducing current space

**Figure 1.**
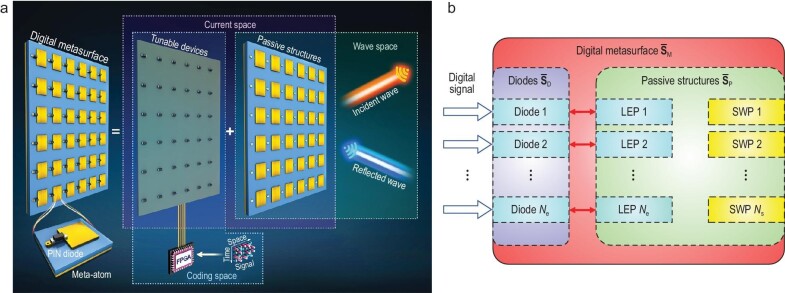
(a) The schematic diagram of a typical digital metasurface, which is divided into three spaces: the coding space, current space and wave space. The three spaces are composed of the digital control signals and tunable devices, the currents on passive structures and tunable devices, and the fields in the region of interest, respectively. (b) The equivalent microwave network of the digital metasurface, in which SWP and LEP are abbreviations of spatial-wave port and lumped-electric port, respectively. The diodes have ${N}_{\mathrm{e}}$ LEPs, the passive structures have ${N}_{\mathrm{e}}$ LEPs and ${N}_{\mathrm{s}}$ SWPs, and the digital metasurface has ${N}_{\mathrm{s}}$ SWPs. The passive structures connect to the diodes through LEPs, and share the SWPs with the digital metasurface.

As shown in Fig. 1(a), the digital metasurface modulates the scattered fields under the control of baseband signals and serves as a platform to link the EM space and the information space. Numerous designs of baseband metasurfaces have been reported in the literature, while the fundamental mechanism is the same, i.e. changing the working states of the meta-atoms by tunable devices such as positive-intrinsic-negative (PIN) and varactor diodes. It is believed that the control voltages imposed on the tunable device directly reflect the states of the meta-atoms. For example, the high and low voltages on a 1-bit meta-atom will induce two states with 180˚ phase difference. However, it should be noted that the scattered field is the integral of the dyadic Green's function and the current over the whole meta-atom, whose domain is larger than that of the current through the tunable devices. The discrete form of this process can be simplified as the multiplication of the channel matrix and transmission vector. Moreover, the mutual coupling prevents the adjacent elements from exhibiting completely opposite states. Consequently, the states of tunable devices or the control voltages imposed on them cannot be equated with those of the scattered fields.

With this in mind, we propose the concept of *current space* as a medium between the digital coding space and the EM wave space. The relationship of the three spaces is shown in Fig. 1(a). The digital metasurface consists of passive structures (dielectrics and metallics) and tunable devices (e.g. PIN diodes). The passive structures are fixed, and they interact with the spatial EM waves and convert them into guided waves. In contrast, the tunable devices do not interact directly with the spatial waves and only modulate the currents in the circuit. Therefore, the digital control voltages and tunable devices form the coding space, while the passive structures and free space form the wave space. The current space consists of the currents on the passive structures and tunable devices, inducing the fields in the wave space. Using the integral operation with the scalar or dyadic Green's function as the kernel, one can easily map the current space to the wave space. Since the integral operation is linear, the mapping from the current space to wave space is linear as well. The relationship between the coding space and the current space has been ignored or regarded as linear in previous studies. However, the relationship is actually non-linear, since the metasurface is a non-linear device with complex effects such as mutual coupling and the imperfect performance of tunable devices. In order to accurately characterize the relationship, we invoke microwave network theory and carry out the analysis.

### Mapping the coding space to the current space

The digital metasurface, the passive structures and the tunable devices can be regarded as three general microwave networks, as shown in Fig. 1(b). Their scattering matrices are denoted as ${\overline {{\bf S}} }_{\mathrm{M}}$, ${\overline {{\bf S}} }_{\mathrm{P}}$ and ${\overline {{\bf S}} }_{\mathrm{D}}$, respectively, where the subscripts represent the metasurface, passive structures and diodes, respectively. In this study, we choose PIN diodes as tunable devices. There are two types of ports in the three networks, namely, the spatial-wave port (SWP) that interacts with the incident and scattered waves, and the lumped-electric port (LEP) that interacts with the guided waves. The passive structures have ${N}_{\mathrm{e}}$ LEPs and ${N}_{\mathrm{s}}$ SWPs, the diodes have ${N}_{\mathrm{e}}$ LEPs, and the digital metasurface has ${N}_{\mathrm{s}}$ SWPs. The passive structures connect to the diodes through the LEPs and share the SWPs with the digital metasurface. The reflected signals at SWPs are the scattered waves of the metasurface, and their states are modified by changing the reflection coefficients of the PIN diodes. ${N}_{\mathrm{e}}$ is the number of tunable devices, which is fixed for a certain metasurface. In contrast, the specific number of ${N}_{\mathrm{s}}$ and the positions of SWPs depend on the definition. In theory, SWPs can be defined at any distance from the metasurface. However, the metasurface does not always work in far-field regions. For example, RISs can be configured to reflect the incident waveform towards a point in the radiative near field, resulting in a beam with finite depth [57]. Therefore, it is more general to predict the near fields and then calculate the fields in the target region. To this end, we define the SWPs in the near-field region of the metasurface.

With the ports of three networks defined, we next analyze their scattering matrices. There is no mutual coupling between diodes, and therefore ${\overline {{\bf S}} }_{\mathrm{D}}$ is a diagonal matrix:


(1) \begin{equation*}
{\overline {{\bf S}} }_{\mathrm{D}} = \left[ {\begin{array}{@{}*{4}{c}@{}} {{\gamma }_1}&\quad 0&\quad \cdots &\quad 0\\ 0&\quad {{\gamma }_2}&\quad \cdots &\quad 0\\ \vdots &\quad \vdots &\quad \ddots &\quad \vdots \\ 0&\quad 0&\quad \cdots &\quad {{\gamma }_{{N}_{\mathrm{e}}}} \end{array}} \right],\end{equation*}

where ${\gamma }_i = \pm 1$ is the reflection coefficient of the PIN diode connected to the *i*th LEP. Based on the types of ports, ${\overline {{\bf S}} }_{\mathrm{P}}$ is composed of four parts:


(2) \begin{equation*}{\overline {{\bf S}} }_{\mathrm{P}} = \left[ {\begin{array}{@{}*{2}{c}@{}} {{{\overline {{\bf S}} }}_{{\mathrm{s,s}}}}&\quad{{{\overline {{\bf S}} }}_{{\mathrm{s,e}}}}\\ {{{\overline {{\bf S}} }}_{{\mathrm{e,s}}}}&\quad{{{\overline {{\bf S}} }}_{{\mathrm{e,e}}}} \end{array}} \right].\end{equation*}

Decomposition in Equation (2) has two advantages: (i) we can separate the scattering behaviors of the passive structures and tunable devices; and (ii) we can effectively describe the mutual coupling between different ports and various non-linear effects by using the microwave network theory. After obtaining the scattering matrices of the passive structures and tunable devices, we invoke the cascade equation in the microwave network theory and calculate ${\overline {{\bf S}} }_{\mathrm{M}}$ as (see the detailed derivations in the Supplementary Materials):


(3) \begin{equation*}{\overline {{\bf S}} }_{\mathrm{M}} = {\overline {{\bf S}} }_{{\mathrm{s,s}}} + {\overline {{\bf S}} }_{{\mathrm{s,e}}}{\overline {{\bf S}} }_{\mathrm{D}}{\left( {{{\overline {{\bf I}} }}_{{N}_{\mathrm{e}}} - {{\overline {{\bf S}} }}_{{\mathrm{e,e}}}{{\overline {{\bf S}} }}_{\mathrm{D}}} \right)}^{ - 1}{\overline {{\bf S}} }_{{\mathrm{e,s}}},\end{equation*}

where ${\overline {{\bf S}} }_{{\mathrm{s,s}}}$, ${\overline {{\bf S}} }_{{\mathrm{s,e}}}$, ${\overline {{\bf S}} }_{{\mathrm{e,e}}}$ and ${\overline {{\bf S}} }_{{\mathrm{e,s}}}$ are the submatrices of ${\overline {{\bf S}} }_{\mathrm{P}}$, ${\overline {{\bf S}} }_{\mathrm{D}}$ is the scattering matrix of the tunable devices, and ${\overline {{\bf I}} }_{{N}_{\mathrm{e}}}$ is the ${N}_{\mathrm{e}}$-dimensional identity matrix. Although the influence of the diodes on the metasurface has been isolated in Equation (3), no direct relationship between the coding space and the current space is obtained. To this end, we approximate the inverse term in Equation (3) as a power series and get the following expression that relates the equivalent current ${\mathrm{J}}$ on the aperture to the reflection coefficients ${\gamma }_i$ of PIN diodes:


(4) \begin{eqnarray*}
&& {\mathop{\rm J}\nolimits} \left( {{\gamma }_1,{\gamma }_2, \ldots ,{\gamma }_{{N}_{\mathrm{e}}},\bar{r}^{\prime}} \right) = {{\mathop{\rm J}\nolimits} }_0\left( {\bar{r}^{\prime}} \right) + \sum\limits_{i = 1}^{{N}_{\mathrm{e}}} {{\mathop{\rm J}\nolimits} _1^i\left( {\bar{r}^{\prime}} \right) \cdot {\gamma }_i}\nonumber\\
&&\quad +\, \sum\limits_{i = 1}^{{N}_{\mathrm{e}} - 1} {\sum\limits_{j = i + 1}^{{N}_{\mathrm{e}}} {{\mathop{\rm J}\nolimits} _1^{i,j}\!\left( {\bar{r}^{\prime}} \right) \cdot {\gamma }_i{\gamma }_j} } \nonumber\\
&&\quad { + \cdots + {\mathop{\rm J}\nolimits} _{{N}_{\mathrm{e}}}^{1,2,3, \cdots ,{N}_{\mathrm{e}}}\left( {\bar{r}^{\prime}} \right) \cdot {\gamma }_1{\gamma }_2 \cdots {\gamma }_{{N}_{\mathrm{e}}}},
\end{eqnarray*}

where the superscript and subscript of ${\rm J}$ indicate the ID (identification number) of the element and the order of current pattern, respectively. The detailed derivations are given in the Supplementary Materials. Here, the zeroth-order term ${{\mathop{\rm J}\nolimits} }_0$ is independent of the state of any meta-atoms and can be regarded as the directly scattered field of the passive structures. The first-order term of ${\gamma }_i$ represents the scattered fields associated with the state of a single meta-atom. The higher-order term of ${\gamma }_i$ represents the scattered fields related to the states of several elements and can be considered the mutual coupling of elements, which is generated by the multiple reflections of the guided waves between LEPs.

In the conventional modeling method, the metasurface is simply equivalent to an array antenna, and the transmission or reflection coefficient obtained by the Floquet-mode simulations is used to describe the scattering performance of meta-atoms. These approximations neglect the mutual coupling and pattern variation, which ultimately gives rise to large errors in the predicted scattered fields of the metasurfaces. From this viewpoint, the conventional modeling method contains only the first-order terms. In contrast, the proposed model here fully accounts for mutual coupling and pattern variation through the higher-order terms in Equation (4), and thus promises an accurate macroscopic model of the digital metasurface.

Equation (4) indicates that the current on the metasurface aperture depends on the coding states of all elements in the array. In order to simplify the equation, we invoke the fact that the coupling between adjacent elements dominates the interaction. Thus we truncate non-adjacent second-order and higher-order terms, obtaining the expression of the current on the (m, n)th element as:


(5) \begin{eqnarray*}
&& {{\mathrm{J}}}^{m,n} = {\mathrm{J}}_0^{m,n} + {\mathrm{J}}_1^{m,n}{s}_{m,n} + {\mathrm{J}}_1^{m - 1,n}{s}_{m - 1,n} \nonumber\\
&&\quad +\, {\mathrm{J}}_1^{m + 1,n}{s}_{m + 1,n} + {\mathrm{J}}_1^{m,n - 1}{s}_{m,n - 1} + {\mathrm{J}}_1^{m,n + 1}{s}_{m,n + 1}\nonumber\\
&&\quad +\, \Big( {{\mathrm{J}}_2^{m - 1,n}{s}_{m - 1,n} + {\mathrm{J}}_2^{m + 1,n}{s}_{m + 1,n} + {\mathrm{J}}_2^{m,n - 1}{s}_{m,n - 1}}\nonumber\\
&&\qquad +\, {{\mathrm{J}}_2^{m,n + 1}{s}_{m,n + 1}} \Big){s}_{m,n} .\end{eqnarray*}

**Figure 2.**
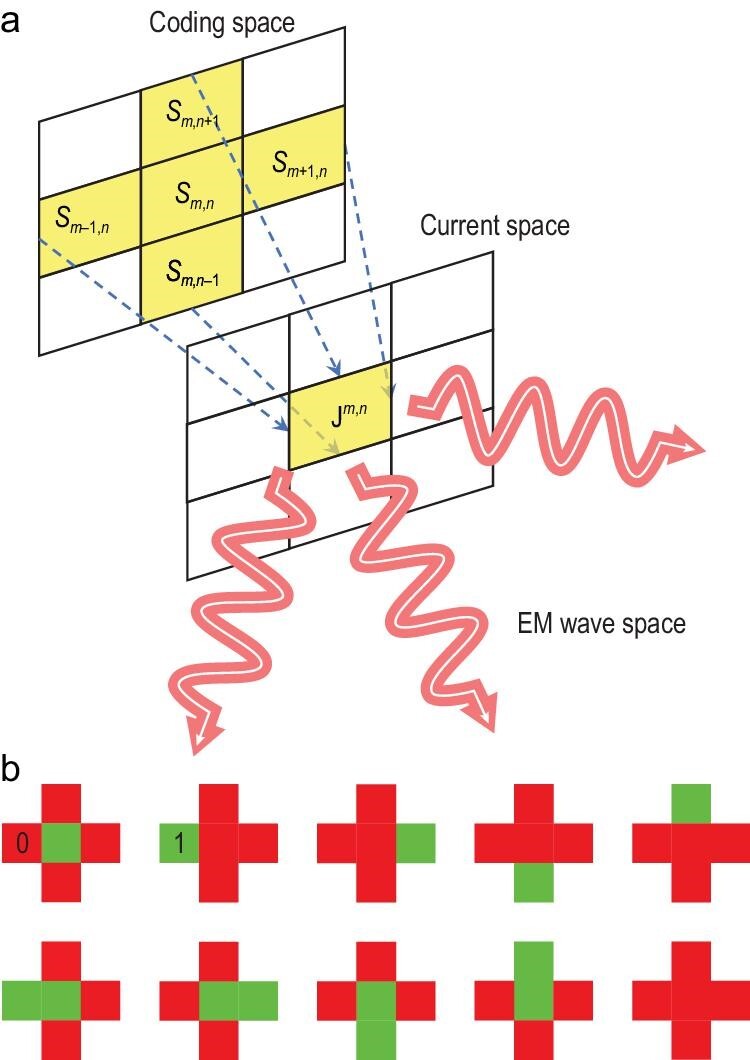
(a) The mapping from the coding space to the current space. The current on the (m, n)th element depends on its coding states and its adjacent elements. (b) Ten coding states are simulated to extract the current patterns of the element. The red and green squares represent ‘0’ and ‘1’ states, respectively.

The superscript and subscript of ${\mathop{\rm J}\nolimits} $ on the right side of Equation (5) indicate the ID of the element and the order of the current pattern, respectively. ${s}_{m,n} = \pm 1$ represents the coding state of the (m, n)th element and is equal to the reflection coefficient of the PIN diode. There are 10 current patterns on one element, including 1 zeroth-order pattern, 5 first-order patterns and 4 second-order patterns. The first-order patterns correspond to the codes of one central and four adjacent elements, while the second-order patterns correspond to the products of the code of the central element and those of the four adjacent ones. Compared to the previous modeling method based on microwave network theory, the proposed method avoids the inverse matrix term in Equation (3). Therefore, the coefficients in Equation (5) are directly multiplied by the coding states. On the other hand, the model separates the influence of adjacent elements on the central element, so we only need a small amount of subarray-level full-wave simulations to extract the current patterns instead of the whole metasurface, which is necessary in the previous method. By this point, a novel and concise macroscopic model linking the coding space and the current space is established, as shown in Fig. 2(a). The key point is that the current on the (m, n)th element depends on its coding states and its neighboring elements, as indicated by the yellow squares.

In summary, the macroscopic model innovatively introduces the concept of current space and includes high-order patterns in the equivalent networks to take the mutual coupling of meta-atoms into consideration. Based on this model, we establish an accurate mapping between the coding space and the current space, and can predict the scattered fields of digital metasurfaces working in any coding states.

The current patterns in the model are complex and there are no analytical expressions, so we use full-wave simulation to extract them. Five 1-bit metasurface elements have 32 coding states in total, but there are only 10 unknowns in Equation (5). Therefore, as long as an appropriate coding sampling scheme is designed, all current patterns can be extracted through 10 full-wave simulations by using the undetermined-coefficients method. The coding states for the 10 sets of simulations are shown in Fig. 2(b), and a detailed explanation is presented in the Supplementary Materials. After obtaining the current patterns, we can use Equation (5) to calculate the equivalent current of the metasurface element in any coding state.

### Simulation and experiment verifications

**Figure 3.**
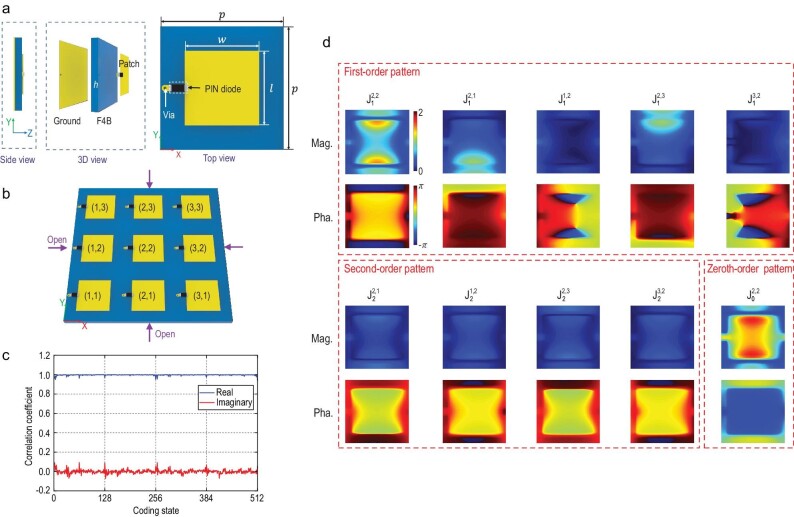
(a) Side, 3D and top views of the 1-bit metasurface element. The working frequency is 5 GHz, and the key parameters are $h = $2 mm, $p = $20 mm, $w = $16.5 mm and $l = $13 mm. (b) The 1-bit metasurface used to verify the accuracy of the macroscopic model. (c) The correlation coefficients between the predicted and simulated equivalent currents. (d) The equivalent current patterns of the (2, 2) element.

To verify the accuracy of the macroscopic model, the metasurface element shown in Fig. 3(a) is designed. It is composed of a rectangle patch printed on a 2 mm-thick substrate (F4B with the dielectric constant of 2.65 and loss tangent of 0.001). A PIN diode (MACOM MADP-000907-14020) is loaded between the patch and the via that is connected to the ground plane. The metasurface element works at a frequency of 5 GHz, and its overall dimension is 1/3 wavelength. The key parameters are $p = $20 mm, $w = $16.5 mm and $l = $ 13 mm. The metasurface in question is shown in Fig. 3(b), which consists of 3 × 3 elements with their ID marked. According to Equation (5), the current on the central element is written as:


(6) \begin{eqnarray*}
{{\mathrm{J}}}^{2,2} &=& {\mathrm{J}}_0^{2,2} + {\mathrm{J}}_1^{2,2}{s}_{2,2} + {\mathrm{J}}_1^{1,2}{s}_{1,2} + {\mathrm{J}}_1^{3,2}{s}_{3,2} + {\mathrm{J}}_1^{2,1}{s}_{2,1} \nonumber\\
&+&\, {\mathrm{J}}_1^{2,3}{s}_{2,3} + \left( {\mathrm{J}}_2^{1,2}{s}_{1,2} + {\mathrm{J}}_2^{3,2}{s}_{3,2} + {\mathrm{J}}_2^{2,1}{s}_{2,1} + {\mathrm{J}}_2^{2,3}{s}_{2,3} \right) {s}_{2,2}. \nonumber\\
\end{eqnarray*}

Full-wave simulations of the metasurface are carried out to extract the 10 current patterns in Equation (6). In simulations, the 10 coding states shown in Fig. 2(b) are impressed on the metasurface under the illumination of *x*-polarized plane waves, and the magnetic fields on the plane, 1 mm in front of the central element, are equivalent to the currents.

The 10 current patterns of the central element are given in Fig. 3(d). We can easily find that ${\mathrm{J}}_0^{2,2}$ and ${\mathrm{J}}_1^{2,2}$ dominate the results, while ${\mathrm{J}}_1^{2,1}$ and ${\mathrm{J}}_1^{2,3}$ take second place. The magnitudes of ${\mathrm{J}}_1^{1,2}$ and ${\mathrm{J}}_1^{3,2}$ are smaller than those of ${\mathrm{J}}_1^{2,1}$ and ${\mathrm{J}}_1^{2,3}$, although they are both first-order patterns of the adjacent coding states. This indicates that the mutual coupling of metasurface elements is different for the two directions. Specifically, the mutual coupling of currents along the *y* direction is stronger than that along the *x* direction. Overall, the magnitudes of the second-order patterns are smaller, being only approximately one-third of that of ${\mathrm{J}}_1^{2,2}$. Once these patterns are obtained, we can substitute any coding state into Equation (6) and then predict the equivalent current on the central element. The 3 × 3 metasurface has 512 coding states in total, and we calculate the correlation coefficients between the predicted and simulated currents and use them as the figure of merits to evaluate the prediction performance of the macroscopic model. The results are given in Fig. 3(c), from which we can see that the proposed model predicts the currents quite well. In particular, most of the correlation coefficients are >0.95. The slight deviation of the correlation coefficients for some of the coding states is due to the truncation of non-adjacent and higher-order currents. We also give the residual sum of squares of predicted currents in the Supplementary Materials. The maximum relative residual is ∼6%, which also proves the accuracy of the proposed prediction model.

**Figure 4.**
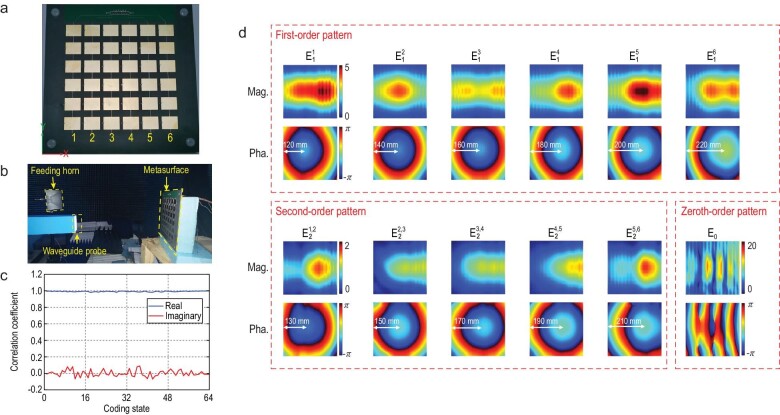
(a) The fabricated 1-bit digital metasurface consists of 6 columns of elements. (b) The near-field measurement scene. (c) The correlation coefficients between the predicted and measured near-fields. (d) The near-field patterns of the metasurface.

Experimental measurements are also carried out to verify the macroscopic model. The fabricated metasurface is shown in Fig. 4(a). It is composed of 6 × 6 meta-elements, which share the same structure, as shown in Fig. 3(a). The difference is that, unlike the full-wave simulations, each column of elements shares the same DC (direct current) feeding. As a result, the metasurface has one zeroth-order pattern, six first-order patterns and five second-order patterns. The measurement scene is shown in Fig. 4(b). The metasurface is excited by a horn antenna in the anechoic chamber, and a waveguide probe is used to pick up the scattered fields. Because the near fields of the reflective metasurface are hard to capture accurately due to the influences of the probe and scanning mechanisms, we measure the electric field on the plane 30 cm in front of the fabricated sample. Though the measurement of the electric fields is not carried out in the near-field region, the data obtained can be used to verify our macroscopic model. The scattered fields of the metasurface are expressed as:


(7) \begin{eqnarray*}
{\mathrm{E}} &=& {{\mathrm{E}}}_0 + {\mathrm{E}}_1^1{s}_1 + {\mathrm{E}}_1^2{s}_2 + {\mathrm{E}}_1^3{s}_3 + {\mathrm{E}}_1^4{s}_4 + {\mathrm{E}}_1^5{s}_5\nonumber\\
&&+\, {\mathrm{E}}_1^6{s}_6 + {\mathrm{ E}}_2^{1,2}{s}_1{s}_2{\mathrm{ + E}}_2^{2,3}{s}_2{s}_3{\mathrm{ + E}}_2^{3,4}{s}_3{s}_4{\mathrm{ + E}}_2^{4,5}{s}_4{s}_5\nonumber\\
&&+\, {\mathrm{E}}_2^{5,6}{s}_5{s}_6,
\end{eqnarray*}

where ${{\mathrm{E}}}_0$, ${\mathrm{E}}_1^m$ and ${\mathrm{E}}_2^{m,n}$ represent the zeroth-order pattern, the first-order pattern of the *m*th column elements and the second-order pattern of the *m*th and *n*th column elements, respectively.

The obtained field patterns are shown in Fig. 4(d). The magnitudes of the second-order patterns are approximately one-third of those of the first-order patterns, and the ratio is consistent with that of the full-wave simulations. The difference in the simulation is that the magnitude of the zeroth-order pattern is much larger than those of the first-order patterns, since the zeroth-order pattern is the sum of all elements’ patterns, and these patterns have propagated over a distance in the space, resulting in a superposition of the fields. We also find that the phase centers of the first-order and second-order patterns are spaced equally at 20 mm intervals, which coincide with the element period. The correlation coefficients between the predicted and measured electric fields are shown in Fig. 4(c), and the curve therein is very close to one, meaning that our model predicts the scattered fields of the metasurface well in the measurement.

## EM INFORMATION ANALYSIS OF THE DIGITAL METASURFACE

EM information theory plays a key role in the design of digital-metasurface-based wireless systems. Though several works on this topic have been reported, the statistical models of digital metasurfaces are based on the conventional methods, which regard the elements as point sources with independent and identically distributed (IID) random amplitudes [27,28]. As mentioned in the previous section, the current on a meta-atom is related to the coding states of several elements and there are non-linear effects, resulting in non-zero current covariance. As a result, the coding space is not equivalent to the current space, and the currents on the metasurface cannot be regarded as IID random point sources.

### Statistical model for digital metasurfaces

Based on the macroscopic model, we further construct a statistical model for the equivalent currents of the digital metasurface. The basic assumption is that coding states obey IID, being the same as the traditional models. However, the probability distributions of element currents will no longer satisfy IID when mutual coupling and other non-linear factors are taken into account by using the proposed macroscopic model.

In typical applications of the digital metasurface, the coding patterns vary as functions of time, and the information is embedded in the scattered fields. Thus, time variables are introduced into the study of the statistical properties of the digital metasurface. For simplicity, we assume that the current patterns are composed of *x*-polarized point sources and the non-tunable zeroth-order pattern can be discarded. In particular, we retain the mutual coupling terms, including first- and second-order patterns of adjacent elements. Hence, the equivalent current on the (m, n)th element can be expressed as:


(8) \begin{eqnarray*}
{{\mathrm{J}}}_{m,n}( t ) &=& {{\mathrm{J}}}_0 \cdot \big\{ {{\mathrm{s}}}_{m,n}( t ) + {c}_{e{\mathrm{,1}}}\big[ {{{\mathrm{s}}}_{m - 1,n}( t ) + {{\mathrm{s}}}_{m + 1,n}( t )} \big]\nonumber\\
&&+\, {c}_{h,1}\big[ {{{\mathrm{s}}}_{m,n - 1}( t ) + {{\mathrm{s}}}_{m,n + 1}( t )} \big] + {c}_{e,2}{{\mathrm{s}}}_{m,n}( t )\nonumber\\
&&\times\, \big[ {{{\mathrm{s}}}_{m - 1,n}( t ) + {{\mathrm{s}}}_{m + 1,n} ( t )} \big] + {c}_{h,2}{{\mathrm{s}}}_{m,n}( t )\nonumber\\
&&\times\, \big[ {{{\mathrm{s}}}_{m,n - 1}( t ) + {{\mathrm{s}}}_{m,n + 1}( t )} \big] \big\} ,
\end{eqnarray*}

in which ${c}_{e,1}$, ${c}_{e,2}$, ${c}_{h,1}$ and ${c}_{h,2}$ represent the first- and second-order coupling coefficients in the E- and H-planes, respectively, and they stand for the ratios of the corresponding current patterns to the first-order pattern of the coding state at the central element; ${{\mathrm{s}}}_{m,n}( t )$ represents the coding state of the (m, n)th element at time *t*, and it can be written as a linear combination of shifted rectangular functions:


(9) \begin{equation*}{{\mathrm{s}}}_{m,n}( t){\mathrm{ = }}\sum\limits_{l = - \infty }^{ + \infty } {{{\mathrm{s}}}_{m,n,l}\Pi \left( {\frac{{t - l{T}_s}}{{{T}_s}}} \right)} ,\end{equation*}

where ${{\mathrm{s}}}_{m,n,l}$ is the coding state of the (m, n)th element at the $l{T}_s$ moment, ${T}_s$ is the symbol duration of the digital signal generated by FPGA (field programmable gate array) or other controllers, and $\Pi ( t )$ is the rectangular function; $\{ {{{\mathrm{s}}}_{m,n,l}} \}$ is an IID two-point random variable sequence with values of {−1, 1}. Correspondingly, the current on the (m, n)th element can also be written as a linear combination of shifted rectangular functions:


(10) \begin{eqnarray*}
{\mathrm{J}}_{m,n}( t) &=& \sum\limits_{l = - \infty }^{ + \infty } {{{\mathrm{J}}}_{m,n,l}\Pi \left( {\frac{{t - l{T}_s}}{{{T}_s}}} \right)}\nonumber \\
&=& {{\mathrm{J}}}_0 \cdot \sum\limits_{l = - \infty }^{ + \infty } \big\{ {{\mathrm{s}}}_{m,n,l} \cdot \big[ {1 + {c}_{{\mathrm{e}},{\mathrm{2}}}( {{{\mathrm{s}}}_{m - 1,n,l} + {{\mathrm{s}}}_{m + 1,n,l}} )}\nonumber\\
&&+ {{c}_{h,2}( {{{\mathrm{s}}}_{m,n - 1,l} + {{\mathrm{s}}}_{m,n + 1,l}} )} \big]
+ {c}_{{\mathrm{e}},{\mathrm{1}}}( {{{\mathrm{s}}}_{m - 1,n,l} + {{\mathrm{s}}}_{m + 1,n,l}} )\nonumber\\
&&+ {c}_{h,1}( {{{\mathrm{s}}}_{m,n - 1,l} + {{\mathrm{s}}}_{m,n + 1,l}} ) \big\} \cdot \Pi \left( {\frac{{t - l{T}_s}}{{{T}_s}}} \right).
\end{eqnarray*}

The frequency spectrum of the current is obtained as:


(11) \begin{eqnarray*}
{\xi }_{m,n}( f) &=& \mathcal{F}[ {{J}_{m,n}( t)} ]\nonumber\\
&=& \mathop {\lim }\limits_{T \to \infty } \frac{1}{{2T}}\int\nolimits_{{ - T}}^{T}{{{J}_{m,n}( t ){e}^{ - i2\pi ft}dt}},\nonumber\\
\end{eqnarray*}

in which the DC component of the spectrum can characterize the capability of the space-time-coding metasurface to modulate the zeroth-order harmonic:


(12) \begin{eqnarray*}
&&\xi _{m,n}^0 = {\xi }_{m,n}( 0 )\nonumber\\
&&\quad=\, {{\mathrm{J}}}_0 \cdot \mathop {\lim }\limits_{L \to \infty } \frac{1}{{2L}}\sum\limits_{l = - L}^L {{{\mathrm{s}}}_{m,n,l}}+ {c}_{{\mathrm{e,1}}} \nonumber\\
&&\quad ( {{{\mathrm{s}}}_{m - 1,n,l} + {{\mathrm{s}}}_{m + 1,n,l}} ) + {c}_{h{\mathrm{,}}1}( {{{\mathrm{s}}}_{m,n - 1,l} + {{\mathrm{s}}}_{m,n + 1,l}} ) \nonumber\\
&&\quad +\, {c}_{{\mathrm{e,2}}} ( {{{\mathrm{s}}}_{m - 1,n,l}{{\mathrm{s}}}_{m,n,l} + {{\mathrm{s}}}_{m + 1,n,l}{{\mathrm{s}}}_{m,n,l}})\nonumber\\
&&\quad + {c}_{h{\mathrm{,}}2} ( {{{\mathrm{s}}}_{m,n - 1,l}{{\mathrm{s}}}_{m,n,l} + {{\mathrm{s}}}_{m,n + 1,l}{{\mathrm{s}}}_{m,n,l}} ).
\end{eqnarray*}

More details are presented in the Supplementary Materials. After denoting:


(13a) \begin{equation*}
{x}_{m,n} \buildrel \Delta \over = \mathop {\lim }\limits_{L \to \infty } \frac{1}{{2L}}\sum\limits_{l = - L}^L {{{\mathrm{s}}}_{m,n,l}}
\end{equation*}

(13b) \begin{equation*}x_{m,n}^e \buildrel \Delta \over = \mathop {\lim }\limits_{L \to \infty } \frac{1}{{2L}}\sum\limits_{l = - L}^L {{{\mathrm{s}}}_{m,n,l}{{\mathrm{s}}}_{m + 1,n,l}} ,\end{equation*}

(13c) \begin{equation*}x_{m,n}^h \buildrel \Delta \over = \mathop {\lim }\limits_{L \to \infty } \frac{1}{{2L}}\sum\limits_{l = - L}^L {{{\mathrm{s}}}_{m,n,l}{{\mathrm{s}}}_{m,n + 1,l}} ,\end{equation*}

we reduce Equation (12) to:


(14) \begin{eqnarray*}
&&\xi _{m,n}^0 = {{\mathrm{J}}}_0 \big[ {x}_{m,n} + {c}_{e,1}\!\left( {{x}_{m - 1,n} + {x}_{m + 1,n}} \right)\nonumber\\
&&+\, {c}_{e,2}\left( {x_{m - 1,n}^e + x_{m,n}^e} \right)+ {c}_{h,1}\!\left( {{x}_{m,n - 1} + {x}_{m,n + 1}} \right)\nonumber\\
&&\, + {c}_{h,2}\!\left( {x_{m,n - 1}^h + x_{m,n}^h} \right) \big].
\end{eqnarray*}

According to the central limit theorem, we know that the mean of massive IID random variables follows the normal distribution, thus:


(15a) \begin{equation*}{x}_{m,n} \sim {\mathrm{N}}( {0,1} ) \end{equation*}

(15b) \begin{equation*}x_{m,n}^e \sim {\mathrm{N}}( {0,1} ),\end{equation*}

(15c) \begin{equation*}x_{m,n}^h \sim {\mathrm{N}}( {0,1}).\end{equation*}

The three random variables ${x}_{m,n}$, $x_{m,n}^e$ and $x_{m,n}^h$ are statistically independent of each other. Since $\xi _{m,n}^0$ is a linear combination of these normally distributed random variables, it is also a normally distributed random variable. Thus, the currents on all elements of the metasurface follow the joint normal distribution, which can be characterized by the covariance matrix. The covariance between any two element currents is given by:


(16a) \begin{equation*}{r}_{(m,n),(m,n)} = 1,\end{equation*}

(16b) \begin{eqnarray*}
{r}_{(m,n),(m + 1,n)} &=& {r}_{1,0}\nonumber\\
&=& \frac{{2{c}_{e,1} + {c}_{e,2}^2}}{{1 + 2{c}_{e,1}^2 + 2{c}_{e,2}^2 + 2{c}_{h,1}^2 + 2{c}_{h,2}^2}},\nonumber\\
\end{eqnarray*}

(16c) \begin{eqnarray*}{r}_{(m,n),(m + 2,n)} &=& {r}_{2,0}\nonumber\\ &=& \frac{{{c}_{e,1}^2}}{{1 + 2{c}_{e,1}^2 + 2{c}_{e,2}^2 + 2{c}_{h,1}^2 + 2{c}_{h,2}^2}},\nonumber\\
\end{eqnarray*}

(16d) \begin{eqnarray*}{r}_{(m,n),(m,n + 1)} &=& {r}_{0,1}\nonumber\\
&=& \frac{{2{c}_{h,1} + {c}_{h,2}^2}}{{1 + 2{c}_{e,1}^2 + 2{c}_{e,2}^2 + 2{c}_{h,1}^2 + 2{c}_{h,2}^2}},\nonumber\\
\end{eqnarray*}

(16e) \begin{eqnarray*}{r}_{(m,n),(m,n + 2)} &=& {r}_{0,2}\nonumber\\
&=& \frac{{{c}_{h,1}^2}}{{1 + 2{c}_{e,1}^2 + 2{c}_{e,2}^2 + 2{c}_{h,1}^2 + 2{c}_{h,2}^2}},\nonumber\\
\end{eqnarray*}

(16f) \begin{eqnarray*}
{r}_{(m,n),(m + 1,n + 1)} &=& {r}_{1,1}\nonumber\\
&=& \frac{{2{c}_{e,1}{c}_{h,1}}}{{1 + 2{c}_{e,1}^2 + 2{c}_{e,2}^2 + 2{c}_{h,1}^2 + 2{c}_{h,2}^2}},\nonumber\\
\end{eqnarray*}

(16g) \begin{eqnarray*}
{r}_{({m}_1,{n}_1),({m}_2,{n}_2)} = 0,\,\,\,{\mathrm{ if }}\left| {{m}_1 - {m}_2} \right| + \left| {{n}_1 - {n}_2} \right| > 2.\nonumber\\
\end{eqnarray*}

More details are presented in the Supplementary Materials.

Finally, for a metasurface consisting of $N \times N$ elements, we can get the joint probability density function of each element current:


(17) \begin{equation*}{\mathrm{p}}\left( {{\bf \xi }} \right) = \frac{1}{{{{\left( {2\pi {{\mathrm{J}}}_{\mathrm{0}}^2} \right)}}^{\frac{{{N}^2}}{2}}{{\left| {{{\overline {{\bf R}} }}_{\left( {N \times N} \right),\left( {N \times N} \right)}} \right|}}^{\frac{1}{2}}}}{e}^{ - \frac{{{{{\bf \xi }}}^{\mathrm{T}} \cdot {{\overline {{\bf R}} }}_{\left( {N \times N} \right),\left( {N \times N} \right)}^{ - 1} \cdot {{\bf \xi }}}}{{2{{\mathrm{J}}}_{\mathrm{0}}^{2{N}^2}}}},\end{equation*}

where ${{\bf \xi }} = {[ {\xi _{1,1}^0,{\mathrm{ }}\xi _{1,2}^0,{\mathrm{ }} \ldots ,{\mathrm{ }}\xi _{N,N}^0} ]}^{\mathrm{T}}$represents the currents on the metasurface aperture, and ${\overline {{\bf R}} }_{( {N \times N} ),( {N \times N} )}$ is the covariance matrix, whose components are given by Equation (16).

**Figure 5.**
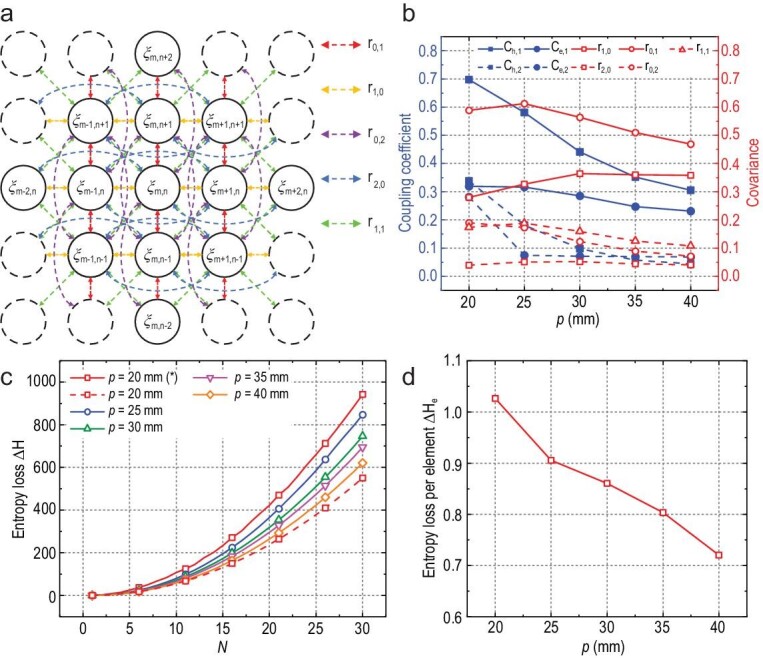
(a) Schematic diagram of the proposed statistical model for the currents on a metasurface. The circles indicate the element currents and the arrows indicate the correlation between two currents. (b) The coupling coefficient and covariance of element currents with different periods. (c) The entropy loss of metasurfaces with different periods and scales. An asterisk indicates that the curve is calculated by the modified model. The modified model is given in the Supplementary Materials. (d) The entropy loss per element.

Through the above analysis, we have transformed the proposed macroscopic model into the statistical model of the currents on the metasurface. The schematic diagram of the proposed statistical model is shown in Fig. 5(a), in which the circles indicate the element currents and the arrows indicate the presence of the correlation between the two currents. Each element current is correlated with the 12 surrounding ones as indicated by the solid circles in Fig. 5(a). The central element and the 12 surrounding ones share the same adjacent element. Therefore, the central current is related to the 12 surrounding ones through the codes of the shared adjacent elements. This leads to non-zero elements in the covariance matrix of the currents. Using the proposed method, we compute the coupling coefficients and the covariance of currents corresponding to the elements with the structure shown in Fig. 3(a) but different spatial periods. The parameters of the metasurface elements are given in the Supplementary Materials. The computational results are illustrated in Fig. 5(b). The curves therein indicate that the coupling coefficient decreases monotonically with the element period, while the variation of covariance is not monotonic. When the coupling coefficient is large enough, the coupling terms dominate the current, resulting in a decrease of the covariance between surrounding elements.

### Current entropy and information loss of the digital metasurface

Knowing the joint probability density function and the covariance matrix of the currents, we can calculate the current entropy of the metasurface:


(18) \begin{eqnarray*}
{\mathrm{H}}\!\left( {{\bf \xi }} \right) &=& - \int{{{\mathrm{p}}\left( {{\bf \xi }} \right)\ln {\mathrm{p}}\left( {{\bf \xi }} \right)d}}{{\bf \xi }}\nonumber\\
&=& \ln\! \left[ {{{\left( {2\pi e{{\mathrm{J}}}_0^2} \right)}}^{\frac{{{N}^2}}{2}}{{\left| {{{\overline {{\bf R}} }}_{\left( {N \times N} \right),\left( {N \times N} \right)}} \right|}}^{\frac{1}{2}}} \right]\nonumber\\
&=& \frac{1}{2}\left( {{N}^2\ln 2\pi e{{\mathrm{J}}}_0^2 + \ln \left| {{{\overline {{\bf R}} }}_{\left( {N \times N} \right),\left( {N \times N} \right)}} \right|} \right).\nonumber\\
\end{eqnarray*}

The first term in the last line of the above expression is a constant and not affected by mutual coupling. The second term only contains the determinant of the covariance matrix; thus, it is 0 when ${\overline {{\bf R}} }_{( {N \times N} ),( {N \times N} )}$ is an identity matrix, otherwise it is negative. We denote the first and second terms ${{\mathrm{H}}}_{\mathrm{0}}$ and $- \Delta {\mathrm{H}}( {{\bf \xi }} )$ respectively. ${{\mathrm{H}}}_{\mathrm{0}}$ represents the information contained in the $N \times N$ IID normally distributed currents, which can also be considered the information possessed by the digital signals in the coding space, while $\Delta {\mathrm{H}}( {{\bf \xi }} )$ represents the entropy loss due to the mutual coupling:


(19) \begin{equation*}\Delta {\mathrm{H}}\!\left( {{\bf \xi }} \right) = {{\mathrm{H}}}_0 - {\mathrm{H}}\!\left( {{\bf \xi }} \right) = - \frac{1}{2}\ln \left| {{{\overline {{\bf R}} }}_{\left( {N \times N} \right),\left( {N \times N} \right)}} \right|.\end{equation*}

Based on the covariance in Fig. 5(b) and Equation (19), we further analyze the information loss from the coding space to the current space for different array sizes and element periods. As shown in Fig. 5(c), the loss of information increases with the size of metasurface. The added elements can be considered as IID sources in digital space. However, mutual coupling prevents the current entropy from reaching the maximum, when digital information is converted into EM information through the metasurface. Therefore, with the increase of array size, the information loss will continue to accumulate. Comparing the curves in Fig. 5(c), we find that entropy loss decreases as the element period increases, consistent with existing studies on digital metasurfaces. However, an anomaly occurs when the period is 20 mm. The reason is that we truncate the higher-order and non-adjacent second-order current patterns when simplifying Equation (4) to Equation (5). The non-zero components of the covariance matrix corresponding to these truncated current patterns cannot be neglected when the element period is small. Therefore, we expand the model to take the influence of diagonal elements on the central element into account. The detailed expanded model is given in the Supplementary Materials. The corrected current entropy loss curve, marked with an asterisk in Fig. 5(c), conforms to the law that information loss decreases with the increase of element period.

We define the average information loss per element as:


(20) \begin{equation*}\Delta {{\mathrm{H}}}_e = \mathop {\lim }\limits_{N \to \infty } \frac{{\Delta {\mathrm{H}}( {{\bf \xi }})}}{{{N}^2}}.\end{equation*}

The variation of the average information loss with respect to the element period is shown in Fig. 5(d). The average entropy loss of element with 20 mm period is calculated based on the curve of the expanded model. Thus, the average entropy loss of metasurface element is a monotonic decreasing function of spatial period.

The proposed statistical model takes full account of mutual coupling between metasurface elements and translates them into a covariance matrix for characterization. Based on the probability density distribution function of the currents, we quantify the information loss in the mapping of the coding space to the current space. This model can be used to evaluate the capacity of digital-metasurface-based wireless communication systems.

## CONCLUSION

In this work, we propose a general macroscopic model for digital metasurfaces, which bridges the digital world and the physical world with the introduction of current space. The model is based on rigorous mathematical and physical analyses, and thus it naturally features several outstanding advantages not exhibited by conventional models:

- it is more accurate and robust since it considers both digital and EM aspects of the metasurface element;
- it has low computational complexity as it leverages the fact that a small amount of simulation or measurement is needed;
- it is general for planar and curved, transmission and reflection, digital metasurfaces and bit antennas with arbitrary bit numbers and polarizations;
- it estimates the scattered fields of metasurface accurately, including both near and far fields.

The feasibility and validity of the proposed macroscopic model are thoroughly demonstrated by full-wave simulations and experimental measurements.

We further develop a statistical model of the currents on the metasurface based on the macroscopic model. This model links the mutual coupling in EM field theory to the covariance in statistics, and describes the capability of the metasurface in transforming digital information. More importantly, the concept of current entropy is proposed in this work based on the macroscopic model and statistical model together, enabling the accurate estimation of the information capacity of digital-metasurface-based wireless communication systems. We believe that the findings of this work are critical for the development of EM information theory, and will facilitate more remarkable advances in both the theory and application of digital metasurfaces beyond 5G (B5G) and 6G systems.

## Supplementary Material

nwad299_Supplemental_File
