# Supplementary material for: Macroscopic model and statistical model to characterize electromagnetic information of a digital coding metasurface
Source: Natl Sci Rev. 2023 Nov 29;11(3):nwad299. doi: 10.1093/nsr/nwad299 (PMC10833471; doi:10.1093/nsr/nwad299)
Supplement: nwad299_Supplemental_File [file nwad299_supplemental_file.docx]

Supplementary Materials for

**Macroscopic model and statistical model to characterize electromagnetic information of digital coding metasurface**

Rui Wen Shao^1,2,†^, Jun Wei Wu^1,2, 3,4,†^, Zheng Xing Wang^1,2^, Hui Xu^1,2^, Han Qing Yang^1,2^, Qiang Cheng^1,2^, and Tie Jun Cui^1,2,3,4^,*

1 Institute of Electromagnetic Space, Southeast University, Nanjing 210096, China;

2 State Key Laboratory of Millimeter Waves, Southeast University, Nanjing 210096, China

3 Peng Cheng Laboratory, Shenzhen, Guangdong 518055, China

4 Pazhou Laboratory (Huangpu), Guangzhou, Guangdong 510555, China

†Equally contributed to this work.

^*^Email: [tjcui@seu.edu.cn](mailto:tjcui@seu.edu.cn)

**1. Detailed derivations of Eq. 3**

The equivalent network of passive structures of the metasurface is described by a scattering matrix , and the equation reads as

$\mathbf{B}=\overline{\mathbf{S}}_{\text{P}}\mathbf{A}$, (S1)

where refer to the incident and reflected waves of the passive structures. According to the types of port, we partition $\mathbf{A}$ and $\mathbf{B}$ as

$\mathbf{A}=\left[\begin{matrix} \mathbf{A}_{\text{s}} \\ \mathbf{A}_{\text{e}} \end{matrix}\right],\text{ }\mathbf{B}=\left[\begin{matrix} \mathbf{B}_{\text{s}} \\ \mathbf{B}_{\text{e}} \end{matrix}\right]$, (S2)

$\left[\begin{matrix} \mathbf{B}_{\text{s}} \\ \mathbf{B}_{\text{e}} \end{matrix}\right]=\left[\begin{matrix} \overline{\mathbf{S}}_{\text{s,s}} & \overline{\mathbf{S}}_{\text{s,e}} \\ \overline{\mathbf{S}}_{\text{e,s}} & \overline{\mathbf{S}}_{\text{e,e}} \end{matrix}\right]\left[\begin{matrix} \mathbf{A}_{\text{s}} \\ \mathbf{A}_{\text{e}} \end{matrix}\right]$, (S3)

where the subscript represents the port type. Similarly, the equivalent network of the metasurface is described by $\overline{\mathbf{S}}_{\text{M}}$, and the equation reads as

$\mathbf{B}_{\text{s}}=\overline{\mathbf{S}}_{\text{M}}\mathbf{A}_{\text{s}}$. (S4)

The passive structures connect with the tunable devices through $N_{\text{e}}$ LEPs. Thus, the incident wave of the tunable devices is $\mathbf{B}_{\text{e}}$ and the reflected wave is $\mathbf{A}_{\text{e}}$,

$\mathbf{A}_{\text{e}}=\overline{\mathbf{S}}_{\text{D}}\mathbf{B}_{\text{e}}$. (S5)

By associating Eq. S3, Eq. S4 and Eq. S5, we get the final scattering matrix $\overline{\mathbf{S}}_{\text{M}}$ of the spatial waves:

$\overline{\mathbf{S}}_{\text{M}}=\overline{\mathbf{S}}_{\text{s,s}}+\overline{\mathbf{S}}_{\text{s,e}}\overline{\mathbf{S}}_{\text{D}}\left(\overline{\mathbf{I}}_{N_{\text{e}}}-\overline{\mathbf{S}}_{\text{e,e}}\overline{\mathbf{S}}_{\text{D}}\right)^{-1}\overline{\mathbf{S}}_{\text{e,s}}$, (S6)

which is Eq. 3 in the main text.

**2. Detailed derivations of Eq. 4**

The inverse term in Eq. 3 is approximated as

$\left(\overline{\mathbf{I}}_{N_{\text{e}}}-\overline{\mathbf{S}}_{\text{e,e}}\overline{\mathbf{S}}_{\text{D}}\right)^{-1}=\overline{\mathbf{I}}_{N_{\text{e}}}+\overline{\mathbf{S}}_{\text{e,e}}\overline{\mathbf{S}}_{\text{D}}+\left(\overline{\mathbf{S}}_{\text{e,e}}\overline{\mathbf{S}}_{\text{D}}\right)^{2}+\left(\overline{\mathbf{S}}_{\text{e,e}}\overline{\mathbf{S}}_{\text{D}}\right)^{3}+\cdots$. (S7)

Hence, we substitute Eq. S7 into Eq. S6 and obtain

$\overline{\mathbf{S}}_{\text{M}}=\overline{\mathbf{S}}_{\text{s,s}}+\overline{\mathbf{S}}_{\text{s,e}}\overline{\mathbf{S}}_{\text{D}}\left[\overline{\mathbf{I}}_{N_{\text{e}}}+\overline{\mathbf{S}}_{\text{e,e}}\overline{\mathbf{S}}_{\text{D}}+\left(\overline{\mathbf{S}}_{\text{e,e}}\overline{\mathbf{S}}_{\text{D}}\right)^{2}+\left(\overline{\mathbf{S}}_{\text{e,e}}\overline{\mathbf{S}}_{\text{D}}\right)^{3}+\cdots\right]\overline{\mathbf{S}}_{\text{e,s}}$. (S8)

The waves reflected by SWPs constitute the main part of the waves scattered by the metasurface. According to EM theory, the field at any position is the combination of these scattered waves. Therefore, the equivalent current $\text{J}$ can be expressed as a linear combination of the elements in $\mathbf{B}_{\text{s}}$, namely,

$\mathrm{J}\left(\gamma_{1},\text{ }\gamma_{2},\text{ }\cdots,\text{ }\gamma_{N_{\text{e}}},\text{ }\bar{r}'\right)=\mathbf{C}^{\text{T}}\mathbf{B}_{\text{s}}$, (S9)

where $\bar{r}'$ represents the position and $\mathbf{C}$ is a constant vector, depending on the definition of LEPs and $\bar{r}'$. By associating Eq. S4, Eq. S8 and Eq. S9, we get

$\mathrm{J}\left(\gamma_{1},\text{ }\gamma_{2},\text{ }\cdots,\text{ }\gamma_{N_{\text{e}}},\text{ }\bar{r}'\right)=\mathbf{C}^{\text{T}}\left\{\overline{\mathbf{S}}_{\text{s,s}}+\overline{\mathbf{S}}_{\text{s,e}}\overline{\mathbf{S}}_{\text{D}}\left[\overline{\mathbf{I}}_{N_{\text{e}}}+\overline{\mathbf{S}}_{\text{e,e}}\overline{\mathbf{S}}_{\text{D}}+\left(\overline{\mathbf{S}}_{\text{e,e}}\overline{\mathbf{S}}_{\text{D}}\right)^{2}+\left(\overline{\mathbf{S}}_{\text{e,e}}\overline{\mathbf{S}}_{\text{D}}\right)^{3}+\cdots\right]\overline{\mathbf{S}}_{\text{e,s}}\right\}\mathbf{A}_{\text{s}}$ (S10)

In the above equation, the matrices $\overline{\mathbf{S}}_{\text{s,s}}$, $\overline{\mathbf{S}}_{\text{s,e}}$, $\overline{\mathbf{S}}_{\text{e,e}}$, $\overline{\mathbf{S}}_{\text{e,s}}$, and the vectors $\mathbf{A}_{\text{s}}$, $\mathbf{C}^{\text{T}}$ depend on the structure, position and excitation of the metasurface and hence they can be combined to become constants. We reduce Eq. S10 to

$\mathrm{J}\left(\gamma_{1},\text{ }\gamma_{2},\text{ }\cdots,\text{ }\gamma_{N_{\text{e}}},\text{ }\bar{r}'\right)=c_{0}+\mathbf{C}_{1}^{\text{T}}\overline{\mathbf{S}}_{\text{D}}\left[\overline{\mathbf{I}}_{N_{\text{e}}}+\overline{\mathbf{S}}_{\text{e,e}}\overline{\mathbf{S}}_{\text{D}}+\left(\overline{\mathbf{S}}_{\text{e,e}}\overline{\mathbf{S}}_{\text{D}}\right)^{2}+\left(\overline{\mathbf{S}}_{\text{e,e}}\overline{\mathbf{S}}_{\text{D}}\right)^{3}+\cdots\right]\mathbf{C}_{2}$, (S11)

where $\mathbf{C}^{\text{T}}\overline{\mathbf{S}}_{\text{s,s}}\mathbf{A}_{\text{s}}$, $\overline{\mathbf{S}}_{\text{s,e}}^{\text{T}}\mathbf{C}$, and $\overline{\mathbf{S}}_{\text{e,s}}\mathbf{A}_{\text{s}}$ are denoted as $c_{0}$, $\mathbf{C}_{1}$, and $\mathbf{C}_{2}$, respectively. The second term in Eq. S11 is the linear combination of the elements in matrix $\overline{\mathbf{S}}_{\text{D}}\left[\overline{\mathbf{I}}_{N_{\text{e}}}+\overline{\mathbf{S}}_{\text{e,e}}\overline{\mathbf{S}}_{\text{D}}+\left(\overline{\mathbf{S}}_{\text{e,e}}\overline{\mathbf{S}}_{\text{D}}\right)^{2}+\left(\overline{\mathbf{S}}_{\text{e,e}}\overline{\mathbf{S}}_{\text{D}}\right)^{3}+\cdots\right]$. Each element of the $\left(\overline{\mathbf{S}}_{\text{e,e}}\overline{\mathbf{S}}_{\text{D}}\right)^{k}$is composed of the *k*th-order terms of $\gamma_{1},\text{ }\gamma_{2},\text{ }\cdots,\text{ }\gamma_{N_{\text{e}}}$. Therefore, the elements of the matrix $\overline{\mathbf{S}}_{\text{D}}\left[\overline{\mathbf{I}}_{N_{\text{e}}}+\overline{\mathbf{S}}_{\text{e,e}}\overline{\mathbf{S}}_{\text{D}}+\left(\overline{\mathbf{S}}_{\text{e,e}}\overline{\mathbf{S}}_{\text{D}}\right)^{2}+\left(\overline{\mathbf{S}}_{\text{e,e}}\overline{\mathbf{S}}_{\text{D}}\right)^{3}+\cdots\right]$ can be written as infinite-order polynomial without constant term. To this end, we collate the coefficients and obtain

$\mathrm{J}\left(\gamma_{1},\text{ }\gamma_{2},\text{ }\cdots,\text{ }\gamma_{N_{\text{e}}},\text{ }\bar{r}'\right)=\sum_{k_{1}=0}^{\infty} \sum_{k_{2}=0}^{\infty} \cdots\sum_{k_{N_{\text{e}}}=0}^{\infty} c'_{k_{1},\text{ }k_{2},\text{ }\cdots,\text{ }k_{N_{\text{e}}}}\left(\prod_{i=1}^{N_{\text{e}}} \gamma_{i}^{k_{i}}\right)$, (S12)

where $c'$ represents the sorted coefficient, and its subscript represents the order of reflection coefficient. The reflection coefficients of ideal PIN diodes are only $\pm1$, hence the high-order terms of $\gamma_{i}$ are

$\gamma_{i}^{k}=\left\{\begin{matrix} \gamma_{i}\text{, if }k\text{ is odd.} \\ 1\text{, if }k\text{ is even.} \end{matrix}\right.$ (S13)

Substituting Eq. S13 into Eq. S12 and collating the coefficients, we get

$$\mathrm{J}\left(\gamma_{1},\text{ }\gamma_{2},\text{ }\cdots,\text{ }\gamma_{N_{\text{e}}},\text{ }\bar{r}'\right)=\mathrm{J}_{0}\left(\bar{r}'\right)+\sum_{i=1}^{N_{\text{e}}} \mathrm{J}_{1}^{i}\left(\bar{r}'\right)\cdot\gamma_{i}+\sum_{i=1}^{N_{\text{e}}-1} \sum_{j=i+1}^{N_{\text{e}}} \mathrm{J}_{1}^{i,j}\left(\bar{r}'\right)\cdot\gamma_{i}\gamma_{j}+\cdots$$

$+\mathrm{J}_{N_{\text{e}}}^{1,2,3,\cdots,N_{\text{e}}}\left(\bar{r}'\right)\cdot\gamma_{1}\gamma_{2}\cdots\gamma_{N_{\text{e}}}$, (S14)

which is Eq. 4 in the main text.

**3. Determining the current patterns in Eq. 5**

We write Eq. 5 in matrix form

$\text{J}^{m,n}=\text{J}_{0}^{m,n}+\mathbf{C}_{\text{J}}^{\text{T}}\mathbf{\Sigma}$, (S15)

where $\mathbf{C}_{\text{J}}=\left[\text{J}_{1}^{m,n},\text{ J}_{1}^{m-1,n},\text{ J}_{1}^{m+1,n},\text{ J}_{1}^{m,n-1},\text{ J}_{1}^{m,n+1},\text{ J}_{2}^{m-1,n},\text{ J}_{2}^{m+1,n},\text{ J}_{2}^{m,n-1},\text{ J}_{2}^{m,n+1}\right]^{\text{T}}$ is the current pattern, and $\mathbf{\Sigma}=\left[s_{m,n},\text{ }s_{m-1,n},\text{ }s_{m+1,n},\text{ }s_{m,n-1},\text{ }s_{m,n+1},\text{ }s_{m-1,n}s_{m,n},\text{ }s_{m+1,n}s_{m,n},\text{ }s_{m,n-1}s_{m,n},\text{ }s_{m,n+1}s_{m,n}\right]^{\text{T}}$ is the coefficient of each pattern. Eq. S15 contains 10 unknowns, which implies that 10 different sets of simulations are needed to solve for them. The coding states for the 10 sets of simulations are shown in Fig. 2(b). Denoting the simulated current patterns and coefficients of each pattern in the $k$th simulation as $\text{J}_{k}$ and $\Sigma_{k}$, respectively, we get the following equation:

$\text{J}_{k}=\text{J}_{0}^{m,n}+\mathbf{C}_{\text{J}}^{\text{T}}\mathbf{\Sigma}_{k},\text{ }k=1,2,\ldots,10$, (S16)

which is non-homogeneous and linear. We use the difference to eliminate the constant term in the equation. Specifically, we subtract the first 9 equations from the last one, getting

$\text{J}_{k}-\text{J}_{10}=\mathbf{C}_{\text{J}}^{\text{T}}\left(\mathbf{\Sigma}_{k}-\mathbf{\Sigma}_{10}\right),\text{ }k=1,2,\ldots,9$. (S17)

Denoting $\text{J}_{k}-\text{J}_{10}$ and $\mathbf{\Sigma}_{k}-\mathbf{\Sigma}_{10}$ as $\Delta\text{J}_{k}$ and $\Delta\mathbf{\Sigma}_{k}$, which are the *k*th column of $\Delta\mathbf{J}$ and $\Delta\overline{\mathbf{\Sigma}}$, we combine the nine equations in Eq. S17 into a matrix equation:

$\Delta\mathbf{J}=\mathbf{C}_{\text{J}}^{\text{T}}\Delta\overline{\mathbf{\Sigma}}$, (S18)

where $\Delta\overline{\mathbf{\Sigma}}=\left[\begin{matrix} 2 & 0 & 0 & 0 & 0 & -2 & -2 & -2 & -2 \\ 0 & 2 & 0 & 0 & 0 & -2 & 0 & 0 & 0 \\ 0 & 0 & 2 & 0 & 0 & 0 & -2 & 0 & 0 \\ 0 & 0 & 0 & 2 & 0 & 0 & 0 & -2 & 0 \\ 0 & 0 & 0 & 0 & 2 & 0 & 0 & 0 & -2 \\ 2 & 2 & 0 & 0 & 0 & 0 & -2 & -2 & -2 \\ 2 & 0 & 2 & 0 & 0 & -2 & 0 & -2 & -2 \\ 2 & 0 & 0 & 2 & 0 & -2 & -2 & 0 & -2 \\ 2 & 0 & 0 & 0 & 2 & -2 & -2 & -2 & 0 \end{matrix}\right]$ is calculated through the 10 coding states in Fig. 2(b). Since $\Delta\overline{\mathbf{\Sigma}}$ is invertible, we directly obtain the expression of the current patterns:

$\mathbf{C}_{\text{J}}^{\text{T}}=\Delta\mathbf{J}\cdot\Delta\overline{\mathbf{\Sigma}}^{-1}$. (S19)

We substitute $\mathbf{C}_{\text{J}}^{\text{T}}$ into Eq. S16 to obtain the zeroth-order pattern:

$\text{J}_{0}^{m,n}=\text{J}_{10}-\Delta\mathbf{J}\cdot\Delta\overline{\mathbf{\Sigma}}^{-1}\mathbf{\Sigma}_{10}$. (S20)

The 10 coding states in Fig. 2(b) ensure $\Delta\overline{\mathbf{\Sigma}}$ full-rank and invertible, however, the chosen ones are not unique. We can also perform more than 10 sets of simulations to determine $\mathbf{C}_{\text{J}}^{\text{T}}$. At this point, Eq. S18 becomes a superdeterminant equation and the inverse matrix $\Delta\overline{\mathbf{\Sigma}}^{-1}$ in Eq. S19 and Eq. S20 becomes pseudo-inverse $\Delta\overline{\mathbf{\Sigma}}^{\text{T}}\left(\Delta\overline{\mathbf{\Sigma}}\cdot\Delta\overline{\mathbf{\Sigma}}^{\text{T}}\right)^{-1}$. This will improve the accuracy of the macroscopic model.

**4. Sum of residual squares of predicted currents**


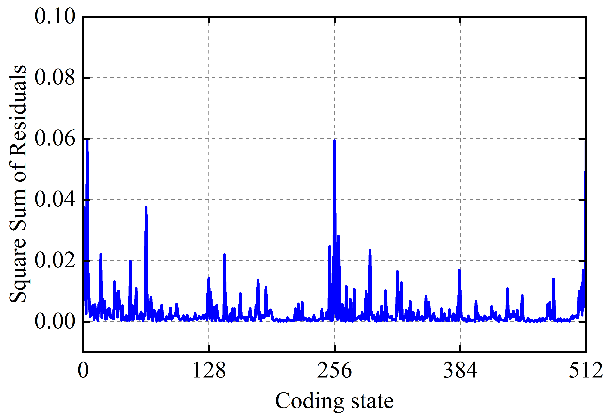


**Figure S1.** Square sum of residuals of the predicted currents.

**5. Detailed derivations of Eq. 12**

Substituting Eq. 10 into Eq. 11, we get:

$$\xi_{m,n}\left(f\right)=\lim_{T\to\infty} \frac{1}{2T}\int_{-T}^{T} J_{m,n}\left(t\right)e^{-i2\pi ft}dt$$

$=\lim_{T\to\infty} \frac{1}{2T}\int_{-T}^{T} \left[\sum_{-\infty}^{\infty} s_{m,n,l}\Pi\left(\frac{t-lT_{s}}{T_{s}}\right)\right]e^{-i2\pi ft}dt$. (S21)

Letting $f=0$, we obtain the DC component:

$\xi_{m,n}^{0}=\xi_{m,n}\left(0\right)=\lim_{T\to\infty} \frac{1}{2T}\int_{-T}^{T} \sum_{-\infty}^{\infty} \text{J}_{m,n,l}\Pi\left(\frac{t-lT_{s}}{T_{s}}\right)\cdot dt$. (S22)

We swap the order of integral and summation symbols in Eq. S22, getting:

$$\begin{matrix} \xi_{m,n}\left(0\right)=\lim_{L\to\infty} \frac{1}{2LT_{\text{s}}}\sum_{l=-L}^{L} \text{J}_{m,n,l}\left(\int_{-LT_{\text{b}}}^{LT_{\text{b}}} \Pi\left(\frac{t-lT_{s}}{T_{\text{s}}}\right)\cdot dt\right) \\ \text{ }=\lim_{L\to\infty} \frac{1}{2L}\sum_{l=-L}^{L} \text{J}_{m,n,l} \\ \text{ }=\text{J}_{0}\cdot\lim_{L\to\infty} \frac{1}{2L}\sum_{l=-L}^{L} \text{s}_{m,n,l}+c_{\text{e，1}}\left(\text{s}_{m-1,n,l}+\text{s}_{m+1,n,l}\right)+c_{h\text{，}1}\left(\text{s}_{m,n-1,l}+\text{s}_{m,n+1,l}\right) \\ \text{ +}c_{\text{e，2}}\left(\text{s}_{m-1,n,l}\text{s}_{m,n,l}+\text{s}_{m+1,n,l}\text{s}_{m,n,l}\right)+c_{h\text{，}2}\left(\text{s}_{m,n-1,l}\text{s}_{m,n,l}+\text{s}_{m,n+1,l}\text{s}_{m,n,l}\right), \end{matrix}$$

(S23)

which is Eq. 12 in the main text.

**6. Detailed derivations of Eq. 16**

Firstly, we calculate the expectation of the product of any two element currents:

(S24a)

, (S24b)

$\text{E}\left\{\xi_{m,n}^{0}\cdot\xi_{m+2,n}^{0}\right\}=\text{J}_{0}^{2}\text{E}\left\{c_{e\text{，}1}^{2}x_{m+1,n}^{2}\right\}=\text{J}_{0}^{2}c_{e\text{，}1}^{2}$, (S24c)

, (S24d)

$\text{E}\left\{\xi_{m,n}^{0}\cdot\xi_{m,n+2}^{0}\right\}=\text{J}_{0}^{2}\text{E}\left\{c_{h\text{，}1}^{2}x_{m,n+1}^{2}\right\}=\text{J}_{0}^{2}c_{h\text{，}1}^{2}$, (S24e)

$\text{E}\left\{\xi_{m,n}^{0}\cdot\xi_{m+1,n+1}^{0}\right\}=\text{J}_{0}^{2}\text{E}\left\{c_{e\text{，}1}c_{h\text{，}1}x_{m+1,n}^{2}+c_{e\text{，}1}c_{h\text{，}1}x_{m,n+1}^{2}\right\}=2\text{J}_{0}^{2}c_{e\text{，}1}c_{h\text{，}1}$, (S24f)

. (S24g)

Then, we can calculate the covariance between any two element currents

$r_{(m,n),(m,n)}=1$, (S25a)

(S25b)

(S25c)

(S25d)

(S25e)

(S25f)

, (S25g)

which is just Eq. 16 in the main text.

**7. Details of the 1-bit metasurface elements**

We design 5 different metasurface elements, whose period is 20 mm, 25 mm, 30 mm, 35 mm, and 40 mm, respectively. They share the similar structure shown in Fig. 3(a), and the specific parameters are shown in Table S1. The full-wave simulated reflection coefficients of these elements are given in Fig. S2(a) and S2(b). The curves show that each of these elements is capable of producing the phase difference of 180 degrees with small amplitude fluctuations at 5 GHz.

**Table S1.** The key parameters of different elements


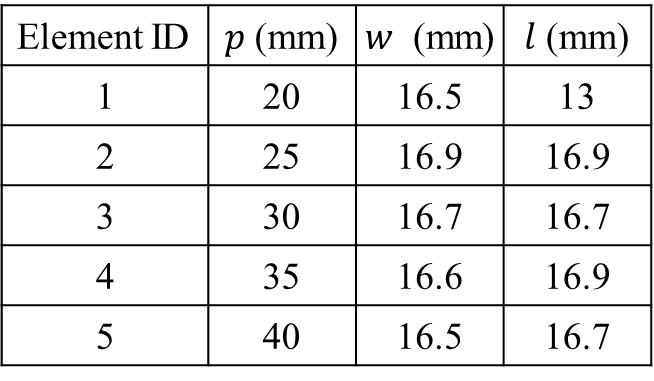


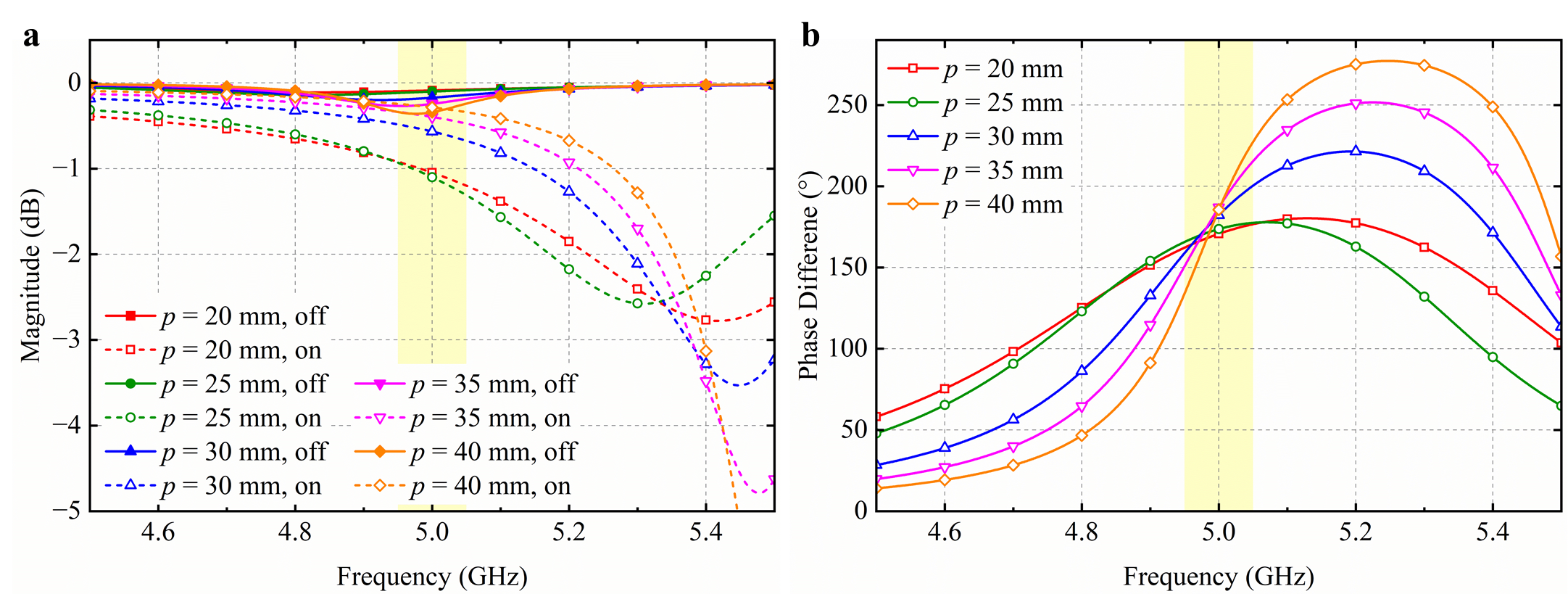


**Figure S2.** (a) The magnitude of the reflection coefficient of elements. (b) The phase of the reflection coefficient of elements.

**8. Expanded statistical model of 20 mm element**

Due to the period is small, the codes of 8 surrounding elements, including 4 adjacent and 4 diagonal ones, will affect the current of the central one. Therefore, Eq. 5 can be modified as

$$\text{J}^{2,2}=\text{J}_{0}^{2,2}+\left(\text{J}_{2}^{1,1}s_{1,1}+\text{J}_{2}^{1,2}s_{1,2}+\text{J}_{2}^{1,3}s_{1,3}+\text{J}_{2}^{2,1}s_{2,1}+\text{J}_{2}^{2,3}s_{2,3}+\text{J}_{2}^{3,1}s_{3,1}+\text{J}_{2}^{3,2}s_{3,2}+\text{J}_{2}^{3,3}s_{3,3}\right)s_{2,2}$$

$+\left(\text{J}_{1}^{1,1}s_{1,1}+\text{J}_{1}^{1,2}s_{1,2}+\text{J}_{1}^{1,3}s_{1,3}+\text{J}_{1}^{2,1}s_{2,1}+\text{J}_{1}^{2,2}s_{2,2}+\text{J}_{1}^{2,3}s_{2,3}++\text{J}_{1}^{3,1}s_{3,1}+\text{J}_{1}^{3,2}s_{3,2}+\text{J}_{1}^{3,3}s_{3,3}\right)$. (S26)

Similarly, Eq. 8 is modified to:

$$\text{J}_{m,n}\left(t\right)\text{=J}_{0}\cdot\left\{\text{s}_{m,n}\left(t\right)+c_{e\text{,1}}\left[\text{s}_{m-1,n}\left(t\right)+\text{s}_{m+1,n}\left(t\right)\right]+c_{h,1}\left[\text{s}_{m,n-1}\left(t\right)+\text{s}_{m,n+1}\left(t\right)\right]\right.$$

$$+c_{e,h\text{,1}}\left[\text{s}_{m-1,n-1}\left(t\right)+\text{s}_{m-1,n+1}\left(t\right)+\text{s}_{m+1,n-1}\left(t\right)+\text{s}_{m+1,n+1}\left(t\right)\right]$$

$$+c_{e,2}\text{s}_{m,n}\left(t\right)\left[\text{s}_{m-1,n}\left(t\right)+\text{s}_{m+1,n}\left(t\right)\right]+c_{h,2}\text{s}_{m,n}\left(t\right)\left[\text{s}_{m,n-1}\left(t\right)+\text{s}_{m,n+1}\left(t\right)\right]$$

$\left.+c_{e,h,2}\text{s}_{m,n}\left(t\right)\left[\text{s}_{m-1,n-1}\left(t\right)+\text{s}_{m-1,n+1}\left(t\right)+\text{s}_{m+1,n-1}\left(t\right)+\text{s}_{m+1,n+1}\left(t\right)\right]\right\}$, (S27)

where two newly added coupling coefficients $c_{e,h,1}=$0.2452 and $c_{e,h,2}=$0.1452 represent the mutual coupling between the central element and the diagonal ones. Then, we calculate the expectation of the product of any two element currents

$\text{E}\left\{\xi_{m,n}^{0}\cdot\xi_{m,n}^{0}\right\}==\text{J}_{0}^{2}\left(1+2c_{e\text{，}1}^{2}+2c_{h\text{，}1}^{2}+2c_{e\text{，}2}^{2}+2c_{h\text{，}2}^{2}+4c_{e,h,1}^{2}+4c_{e,h,2}^{2}\right)$, (S28a)

$\text{E}\left\{\xi_{m,n}^{0}\cdot\xi_{m+1,n}^{0}\right\}=\text{J}_{0}^{2}\left(2c_{e\text{，}1}+c_{e\text{，}2}^{2}+4c_{h,1}c_{e,h,1}\right)$, (S28b)

$\text{E}\left\{\xi_{m,n}^{0}\cdot\xi_{m+2,n}^{0}\right\}=\text{J}_{0}^{2}\left(c_{e\text{，}1}^{2}+2c_{e,h,1}^{2}\right)$, (S28c)

$\text{E}\left\{\xi_{m,n}^{0}\cdot\xi_{m,n+1}^{0}\right\}=\text{J}_{0}^{2}\left(2c_{h\text{，}1}+c_{h\text{，}2}^{2}+4c_{e,1}c_{e,h,1}\right)$, (S28d)

$\text{E}\left\{\xi_{m,n}^{0}\cdot\xi_{m,n+2}^{0}\right\}=\text{J}_{0}^{2}\left(c_{h\text{，}1}^{2}+2c_{e,h,1}^{2}\right)$, (S28e)

$\text{E}\left\{\xi_{m,n}^{0}\cdot\xi_{m+1,n+1}^{0}\right\}=\text{J}_{0}^{2}\left(2c_{e\text{，}1}c_{h\text{，}1}+2c_{e,h,1}+c_{e,h,2}^{2}\right)$, (S28f)

$\text{E}\left\{\xi_{m,n}^{0}\cdot\xi_{m+1,n+2}^{0}\right\}=2\text{J}_{0}^{2}c_{h,1}c_{e,h,1}$, (S28g)

$\text{E}\left\{\xi_{m,n}^{0}\cdot\xi_{m+2,n+1}^{0}\right\}=2\text{J}_{0}^{2}c_{e,1}c_{e,h,1}$, (S28h)

$\text{E}\left\{\xi_{m,n}^{0}\cdot\xi_{m+2,n+2}^{0}\right\}=\text{J}_{0}^{2}c_{e,h,1}^{2}$, (S28i)

. (S28j)

Finally, we can calculate the modified covariance between any two element currents

$r_{(m,n),(m,n)}=1$, (S30a)

$r_{(m,n),(m+1,n)}=r_{1,0}=\frac{2c_{e\text{，}1}+c_{e\text{，}2}^{2}+4c_{h,1}c_{e,h,1}}{1+2c_{e\text{，}1}^{2}+2c_{e\text{，}2}^{2}+2c_{h\text{，}1}^{2}+2c_{h\text{，}2}^{2}+4c_{e,h,1}^{2}+4c_{e,h,2}^{2}}$, (S30b)

$r_{(m,n),(m+2,n)}=r_{2,0}=\frac{c_{e\text{，}1}^{2}+2c_{e,h,1}^{2}}{1+2c_{e\text{，}1}^{2}+2c_{e\text{，}2}^{2}+2c_{h\text{，}1}^{2}+2c_{h\text{，}2}^{2}+4c_{e,h,1}^{2}+4c_{e,h,2}^{2}}$, (S30c)

$r_{(m,n),(m,n+1)}=r_{0,1}=\frac{2c_{h\text{，}1}+c_{h\text{，}2}^{2}+4c_{e,1}c_{e,h,1}}{1+2c_{e\text{，}1}^{2}+2c_{e\text{，}2}^{2}+2c_{h\text{，}1}^{2}+2c_{h\text{，}2}^{2}+4c_{e,h,1}^{2}+4c_{e,h,2}^{2}}$, (S30d)

$r_{(m,n),(m,n+2)}=r_{0,2}=\frac{c_{h\text{，}1}^{2}+2c_{e,h,1}^{2}}{1+2c_{e\text{，}1}^{2}+2c_{e\text{，}2}^{2}+2c_{h\text{，}1}^{2}+2c_{h\text{，}2}^{2}+4c_{e,h,1}^{2}+4c_{e,h,2}^{2}}$, (S30e)

$r_{(m,n),(m+1,n+1)}=r_{1,1}=\frac{2c_{e\text{，}1}c_{h\text{，}1}+2c_{e,h,1}+c_{e,h,2}^{2}}{1+2c_{e\text{，}1}^{2}+2c_{e\text{，}2}^{2}+2c_{h\text{，}1}^{2}+2c_{h\text{，}2}^{2}+4c_{e,h,1}^{2}+4c_{e,h,2}^{2}}$, (S30f)

$r_{(m,n),(m+1,n+2)}=r_{1,2}=\frac{2c_{h,1}c_{e,h,1}}{1+2c_{e\text{，}1}^{2}+2c_{e\text{，}2}^{2}+2c_{h\text{，}1}^{2}+2c_{h\text{，}2}^{2}+4c_{e,h,1}^{2}+4c_{e,h,2}^{2}}$, (S30g)

$r_{(m,n),(m+2,n+1)}=r_{2,1}=\frac{2c_{e,1}c_{e,h,1}}{1+2c_{e\text{，}1}^{2}+2c_{e\text{，}2}^{2}+2c_{h\text{，}1}^{2}+2c_{h\text{，}2}^{2}+4c_{e,h,1}^{2}+4c_{e,h,2}^{2}}$, (S30h)

$r_{(m,n),(m+2,n+2)}=r_{2,2}=\frac{c_{e,h,1}^{2}}{1+2c_{e\text{，}1}^{2}+2c_{e\text{，}2}^{2}+2c_{h\text{，}1}^{2}+2c_{h\text{，}2}^{2}+4c_{e,h,1}^{2}+4c_{e,h,2}^{2}}$, (S30i)

. (S30j)
